# A role for hippocampal PSA-NCAM and NMDA-NR2B receptor function in flavonoid-induced spatial memory improvements in young rats

**DOI:** 10.1016/j.neuropharm.2013.12.003

**Published:** 2014-04

**Authors:** Catarina Rendeiro, Andrew Foley, Vera C. Lau, Rebecca Ring, Ana Rodriguez-Mateos, David Vauzour, Claire M. Williams, Ciaran Regan, Jeremy P.E. Spencer

**Affiliations:** a Molecular Nutrition Group, School of Chemistry, Food and Pharmacy, University of Reading, Reading RG6 6AP, UK; b School of Psychology and Clinical Language Sciences, University of Reading, Reading RG6 6AL, UK; c Berand Neuropharmacology, NovaUCD, Belfield Innovation Park, UCD, Belfield, Dublin 4, Ireland; d School of Biomolecular and Biomedical Science, UCD Conway Institute, University College Dublin, Belfield, Dublin 4, Ireland

**Keywords:** Memory, Learning, Flavonoid, Hippocampus, BDNF, PSA-NCAM, NMDR2B

## Abstract

The increase in incidence and prevalence of neurodegenerative diseases highlights the need for a more comprehensive understanding of how food components may affect neural systems. In particular, flavonoids have been recognized as promising agents capable of influencing different aspects of synaptic plasticity resulting in improvements in memory and learning in both animals and humans. Our previous studies highlight the efficacy of flavonoids in reversing memory impairments in aged rats, yet little is known about the effects of these compounds in healthy animals, particularly with respect to the molecular mechanisms by which flavonoids might alter the underlying synaptic modifications responsible for behavioral changes. We demonstrate that a 3-week intervention with two dietary doses of flavonoids (*Dose I*: 8.7 mg/day and *Dose II*: 17.4 mg/day) facilitates spatial memory acquisition and consolidation (24 recall) (*p* < 0.05) in young healthy rats. We show for the first time that these behavioral improvements are linked to increased levels in the polysialylated form of the neural adhesion molecule (PSA-NCAM) in the dentate gyrus (DG) of the hippocampus, which is known to be required for the establishment of durable memories. We observed parallel increases in hippocampal NMDA receptors containing the NR2B subunit for both 8.7 mg/day (*p* < 0.05) and 17.4 mg/day (*p* < 0.001) doses, suggesting an enhancement of glutamate signaling following flavonoid intervention. This is further strengthened by the simultaneous modulation of hippocampal ERK/CREB/BDNF signaling and the activation of the Akt/mTOR/Arc pathway, which are crucial in inducing changes in the strength of hippocampal synaptic connections that underlie learning. Collectively, the present data supports a new role for PSA-NCAM and NMDA-NR2B receptor on flavonoid-induced improvements in learning and memory, contributing further to the growing body of evidence suggesting beneficial effects of flavonoids in cognition and brain health.

## Highlights

• We have identified novel targets for the flavonoid actions in the brain.
• Flavonoid ingestion facilitates hippocampal-dependent learning in healthy animals.
• Flavonoids regulate hippocampal PSA-NCAM in the Dentate Gyrus of the hippocampus.
• Flavonoids modulate the levels of hippocampal glutamate receptors (NMDA-NR2B).

## 1 Introduction

Physical exercise and a diet rich in fruit and vegetables are known to strongly influence the prevalence, and onset of, cardiovascular disease and neurological disorders (Kromhout et al., 2002; Parrott and Greenwood, 2007). In particular, foods and beverages such as blueberries, green tea and cocoa, which are a group of plant-derived foods/beverages rich in a group of polyphenols called flavonoids, have been shown to have a positive impact on memory, learning and cognitive function in both animals and humans (Dinges, 2006; Haque et al., 2006; Kaur et al., 2008; Krikorian et al., 2010; Williams et al., 2008). The mechanisms by which flavonoids exert these actions on cognitive performance are being elaborated with evidence suggesting that they may modulate the activation status of neuronal receptors, signaling proteins and gene expression (Schroeter et al., 2007; Spencer, 2008; Vauzour et al., 2007). For example, the effects of green tea (Li et al., 2009a,b) and blueberry (Williams et al., 2008) on spatial memory have been shown to involve activation/phosphorylation of the ERK and PI3 kinase/Akt pathways, increased CREB phosphorylation and elevated BDNF levels in the hippocampus.

Such signaling pathways regulate downstream changes in receptor density and cell adhesion molecule expression/recruitment, crucial events in the regulation of various aspects of synaptic function (Cull-Candy et al., 2001; Muller et al., 2000; Sheng and Kim, 2002). With regards to the former, NMDA receptors (NMDA-R) and AMPA receptors (AMPA-R) are key mediators of excitatory synaptic transmission in the hippocampus, and their regulation is regarded as vital for the induction and maintenance of LTP (Cull-Candy and Leszkiewicz, 2004; Kim et al., 2005; Li and Keifer, 2009). In particular, NMDAR-dependent Ca^2+^ influx triggers activation of MAPK kinases, such as ERK, leading to persistent changes in the expression of AMPA receptors (MacDonald et al., 2006), which when trafficked to synaptic sites are known to have an important role in synaptic plasticity and consequently in memory and learning (Malinow and Malenka, 2002; Rumpel et al., 2005). For example, AMPA-class glutamate receptors stabilize spine morphology (Li and Keifer, 2009), whilst an increase in the density of specific NMDA receptor subunits profoundly affects NMDAR-dependent LTP, the major cellular mechanism underpinning spatial learning (Cull-Candy et al., 2001; Cull-Candy and Leszkiewicz, 2004; Perez-Otano and Ehlers, 2004). Flavonoid-rich foods, such as blueberry, have previously been shown to regulate receptors involved in hippocampal plasticity, notably IGF-1 and its receptor (Casadesus et al., 2004) and levels of NMDAR subunits (Coultrap et al., 2008).

In addition to changes in receptor density, cell adhesion molecules such as the polysialylated form of the neural cell adhesion molecule (PSA-NCAM), are recognized as having an important role in activity-dependent changes in synapse strength and morphology (Benson et al., 2000; Ronn et al., 2000, 1998), in defining memory and learning processes (Aonurm-Helm et al., 2008; Kolkova et al., 2000; Muller et al., 2000) and are known to be linked to NMDAR activation (Bouzioukh et al., 2001; Butler et al., 1999). PSA-NCAM is enriched at postsynaptic sites where it modulates synaptic transmission (Hammond et al., 2006; Kiss et al., 2001; Kochlamazashvili et al., 2012; Muller et al., 1996) and is required for the establishment of durable memories (Becker et al., 1996; Doyle et al., 1992; Lopez-Fernandez et al., 2007; Seymour et al., 2008). In view of this, in the present study we have investigated the impact of a ‘dietary-level’ flavonoid intervention on the expression of PSA-NCAM and NMDAR in the hippocampus and how these changes may control, or be controlled by signaling cascades previously observed to change in response flavonoid interventions (Williams et al., 2008). Our data suggest that a 3-week flavonoid administration is capable of enhancing PSA-NCAM levels in the DG along with increases in the NMDA receptor subunit NR2B and that these changes appear to be related to changes in BDNF-related signaling pathways.

## 2 Materials and methods

### 2.1 Materials

Antibodies used were anti-ACTIVE MAPK (ERK1/2), ERK1/ERK2, anti-CREB, anti-GAPDH, anti-phospho-Akt (Ser^473^), anti-Akt, anti-phospho-mTOR (Ser^2448^), anti-phospho-mTOR (Ser^2481^), anti-mTOR, anti-NMDAR1, anti-NMDAR2B, anti-NMDAR2A, anti-AMPA (GluR1/2/3), anti-TrkB (New England Biolabs, Hitchin, UK); anti-BDNF, anti-Arc/Arg3.1 (Santa Cruz Biotechnology, Santa Cruz, CA); anti-CREB (Ser^133^), anti-pro-BDNF, (Millipore, Warford, UK) and anti-PSA monoclonal antibody (Chemicon, UK). Horseradish peroxidase-conjugated goat anti-rabbit secondary antibody (Sigma, UK), ECL reagent and Hyperfilm-ECL were purchased from Amersham Biosciences (Amersham, UK) and FITC-conjugated goat anti-mouse IgM from Calbiochem, UK. HPLC-grade hexane, acetone, glacial acetic, acetonitrile, methanol, water, and hydrochloric acid were purchased from Fischer Scientific (Loughborough, UK). All other reagents were obtained from Sigma or Merck (Poole, UK).

### 2.2 Intervention diets and animal supplementation

Three groups of 8-week old experimentally naïve adult male Wistar rats (*n* = 8 per group, Harlan UK) were housed in groups of four and maintained on a 12 h light–dark cycle (lights on at 10 a.m.) with *ad libitum* access to food and water. Prior to all experiments, animals were introduced into the experimental room for a period of at least 1 h. The flavonoids (both anthocyanins and flavanols) were delivered/administered using a fully characterized blueberry powder (Table 1) from *Vaccinium Corymbosum* high-bush blueberries (A.G. Axon and Sons, UK). The powder was dissolved in water and administered daily to each rat individually by oral gavage (twice a day). Animals were administered daily by oral gavage either *Dose I*: 8.7 mg/day/animal of total flavonoids (anthocyanins: 5.37 mg/day and flavanols: 3.34 mg/day), similar to that used previously (Joseph et al., 1999; Williams et al., 2008), or *Dose II*: 17.4 mg of total flavonoids (anthocyanins: 10.75 mg/day and flavanols: 6.67 mg/day), or a macro- and micro-nutrient control (e.g. equal vitamin C, fructose, glucose, sucrose), for a total of 3 weeks (2 weeks prior spatial testing and 1 week during testing). Both doses administered reflect dietary amounts that can be achieved easily through diet. Analysis and quantification of flavonoids prior to the intervention (flavanols and anthocyanins) was performed by HPLC with diode array detection as previously described (Rodriguez-Mateos et al., 2012) and indicated that the powder contained approximately 179.12 mg of anthocyanins/100 g FW (fresh blueberry weight) and 111.12 mg of flavanols/100 g FW.

### 2.3 Water maze training

The water maze task was carried out as previously described (Murphy et al., 1996). Briefly, the water maze apparatus consisted of a circular pool (1.6 m in diameter, 80 cm high, temperature 26 °C) with a platform (11 cm diameter) submerged 1.5 cm beneath the surface. Both pool and platform were constructed from black Perspex and offered no intra-maze cues to guide escape behavior. The testing room offered several strong extra-maze visual cues to aid the formation of a spatial map. The spatial memory acquisition task consisted of 4 sessions (1 session a day for 4 consecutive days). Each session consisted of 5 testing trials in which animals attempt to locate a hidden platform. Trials were initiated with each rat facing the wall of the maze at one of three designated locations after which animals were allowed to explore the water maze and the time taken to locate the hidden platform was defined as the escape latency. During each test session, the platform was hidden at the same location in the same quadrant 30 cm from the sidewall and animals were free to explore the maze for a period of 60 s, with those failing to locate the platform within this period placed on it for 10 s. Escape latencies were measured over four days of testing with five trials in each session and an inter-trial rest interval of 300 s, during which animals were dried off and returned to the holding cage. The performance at each session is measured as an average of the escape latencies of the 5 testing trials within that session. Swim behavior in the water maze paradigm was monitored using Watermaze 3.1, a Labview^®^ executable image motion analyzer written by Matthias Grossmann (Dresden, Germany) linked to a CCD camera via an image acquisition card (IMAQ-1408, National Instruments Co., UK). A probe trial was performed 24 h after the 4th and final training session. During the probe trial animals were returned to the water maze for 30 s in which the platform had been removed and the amount of time spent in the quadrant that originally contained the platform was recorded, as was the time spent in the other three quadrants.

At the end of the last testing session animals were sacrificed by decapitation and the brains were immediately extracted and halved. Half of the brain was coated in optimum cutting temperature (OCT) compound and lowered into a Cryoprep freezing apparatus containing dry-ice-cooled n-hexane and used for NCAM-PSA immunolabeling experiments (the combination of the OCT and n-hexane was used to ensure even freezing of the tissue to avoid freezing artifacts). From the other half of the brain, the hippocampus was dissected and frozen at −80 °C until use for Western immunoblotting analysis. All experimental procedures were approved by the Animal Research Ethics Committee of University College Dublin, conformed to EU Council Directive 86-609-EEC, and were carried out by individuals retaining the appropriate license issued by the Irish Department of Health.

### 2.4 Preparation of brain sections and PSA-NCAM immunolabeling

The immunohistochemical procedures employed to detect NCAM PSA have been described in greater detail previously (Fox et al., 1995). Briefly, horizontal frozen brain sections (12 μm) were cut on a Microm Series 500 cryostat at −15 °C (*n* = 4 for the Control group, *n* = 5 for the Dose II group). All sections were prepared on the day of the experiment and were not pre-cut and stored frozen. For the analysis of the NCAM PSA-positive hippocampal dentate granule cell layer/hilus border cells, 10 alternate sections were taken at a level equivalent to −5.6 mm below bregma (Paxinos and Watson, 1986). Cryosections were thaw-mounted onto glass slides and immersion fixed for 30 min with 70% (v/v) ethanol and incubated overnight with anti-PSA monoclonal antibody diluted 1:500 in 0.1M PBS containing 1% (w/v) bovine serum albumen (BSA) and 1% (v/v) normal goat serum (NGS). The sections were exposed for 3 h to FITC-conjugated goat anti-mouse IgM diluted 1:100, again in PBS containing 1% BSA and 1% NGS, and mounted in Citifluor (Agar, UK).

### 2.5 Quantitative evaluation of PSA-NCAM expression

Quantitative image analysis was performed using a Leica Quantimet 500 PC-based software package connected to a Leitz DM RB fluorescent microscope with a high sensitivity CCD video camera. Each microscope lens was calibrated for length and area measurements using a 1 mm graticule. The total number of NCAM PSA-immunoreactive neurons in the dentate granule cell layer/hilar border was counted in 6 alternate 12 μm sections commencing −5.6 mm from bregma (Paxinos and Watson, 1986), to preclude double counting of the 5–10 μm perikarya. Cell identification was aided by the use of the nuclear counter-stain propidium iodide (40 ng/ml PBS; 60 s). Cell counts were standardized to unit area of the granule cell layer, 0.15 ± 0.01 mm^2^ at this level, and expressed as mean ± SEM values.

### 2.6 Western immunoblotting

Dissected hippocampus were homogenized on ice with a glass homogenizer using Tris (50 mM), Triton X-100 (0.1%), NaCl (150 mM) and EGTA/EDTA (2 mM); pH 7.4, containing mammalian protease inhibitor cocktail (1:100 dilution), sodium pyrophosphate (1 mM), PMSF (10 μg/ml), sodium vanadate (1 mM) and sodium fluoride (50 mM). Homogenates were left on ice for 45 min before centrifugation at 1000 × g for 5 min at 4 °C to remove unbroken cell debris and nuclei. For analysis of proteins by Western immunoblotting, samples were incubated for 2 min at 95 °C in boiling buffer (final concentration 62.5 mM Tris, pH6.8; 2% SDS; 5% 2-mercaptoethanol; 10% glycerol and 0.0025% bromophenol blue) and stored at −80 °C until analysis. Protein samples (40–80 mg/lane) were run on 9–12% SDS-polyacrylamide gels and proteins were transferred to nitrocellulose membranes (Hybond-ECL^®^; Amersham) by semi-dry electroblotting (1.5 mA/cm^2^). The nitrocellulose membrane was then incubated in a blocking buffer (20 mM Tris, pH 7.5, 150 mM NaCl; TBS) containing 4% (w/v) skimmed milk powder for 45 min at room temperature followed by 2 × 5 min washes in TBS supplemented with 0.05% (v/v) Tween 20 (TTBS). Blots were then incubated overnight at room temperature on a three dimensional rocking table with either anti-BDNF pAb (1:1000), anti-pro-BDNF pAb (1:1000), anti-phospho CREB (Ser^133^) pAb (1:500), anti-CREB (1:1000), anti-ACTIVE MAPK pAb (1:1000 dilution), anti-ERK1/ERK2 pAb (1:1000), anti-phospho-Akt (Ser^473^) pAb (1:1000), anti-Akt pAb (1:1000), anti-phospho-mTOR (Ser^2448^) pAb (1:2000), anti-phospho-mTOR (Ser^2481^) (1:2000), anti-mTOR pAb (1:5000), anti-Arc/Arg3.1 pAb (1:5000), anti-NMDAR1 (1:1000), anti-NMDAR2B (1:1000), anti-NMDAR2A (1:1000), anti-AMPA (GluR1/2/3) (1:1000), anti-TrkB (1:1000) or anti-GAPDH pAb (1:5000), in TTBS containing 1% (w/v) skimmed milk powder (antibody buffer). The blots were washed 2 × 10 min in TTBS and incubated with goat anti-rabbit IgG conjugated to HRP (1:1000 dilution) for 60 min. Finally blots were washed 2 × 10 min in TTBS rinsed in TBS and exposed to ECL-reagent^®^ for 1–2 min and developed. Bands were analyzed using the band analysis software UVISoft Band. Molecular weights of the bands were calculated from comparison with pre-stained molecular weight markers (MW 27,000–180,000 and MW 6500–45,000, BioRad) that were run in parallel with the samples. Relative band intensities were calculated as a ratio of the phosphorylated protein to total protein in the case of ERK1/2, Akt, CREB, mTOR. For BDNF, pro-BDNF, Arc, NMDR1, NMDR2B, NMDAR2A, TrkB, AMPA relative band intensity was calculated by comparison with GAPDH levels.

### 2.7 Statistics

The behavioral data was analyzed using a repeated measures 3-way analysis of variance (ANOVA) with Session, Trial and Treatment as main factors. For immunoblot data, statistical comparisons between the three treatment groups were carried out using a one-way ANOVA. Tukey Post-hoc tests were subsequently used to examine differences between the individual treatments at a confidence level of 95%. Student's *t* test was used for the PSA-NCAM immunolabeling experiments. Correlation coefficients between memory scores and the markers synaptic plasticity, PSA-NCAM, NMDA-NR2B and Arc, were calculated using the Pearson product–moment correlation coefficient. All the data is expressed as mean (±S.E.M) and was analyzed using SPSS.

## 3 Results

### 3.1 Spatial working memory

**Fig. 1:**
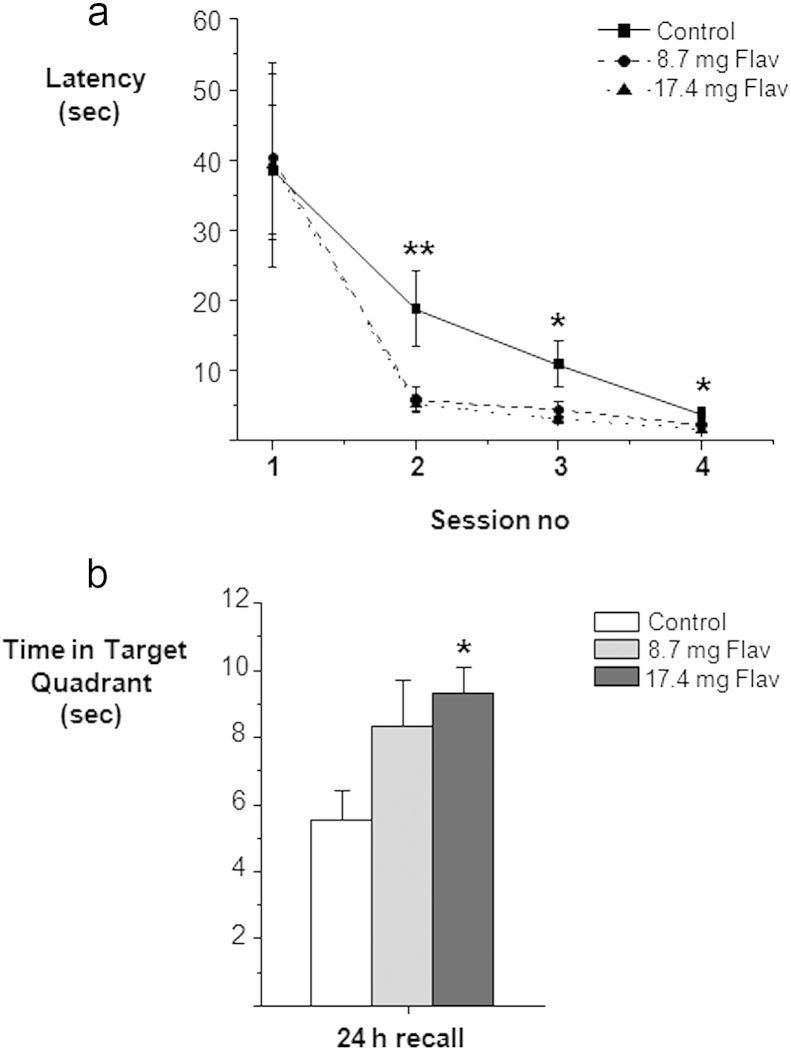
Effects of 3 weeks Flavonoid supplementation on spatial memory in young rats. (a) Effect of flavonoid supplementation on acquisition of the Morris Water Maze task was measured as latency to find the platform (sec). There is a significant decrease in escape latency for both 8.7 mg and 17.4 mg of flavonoids in comparison to control for session 2 (**p < 0.01, *n* = 8), session 3 and 4 (*p < 0.05, *n* = 8), indicating a faster acquisition of the task by the flavonoid groups. (b) 24 h recall of spatial memory measured as time spent in target quadrant. Only the 17.4 mg flavonoid group showed significantly enhanced recall of the platform location (*p < 0.05, *n* = 8). The 8.7 mg flavonoid group showed a trend for increase relative to control animals (*p* = 0.1).

As anticipated, young rodents showed a significant increase in weight over the time course of the experiment (*F* 27, 567 = 70.425, *p* < 0.001). However, the increase in weight was similar for all three experimental groups (control: 6.3%; 8.7 mg Flav: 5.9%; 17.4 mg Flav : 6.5%) and, there was no significant effect of treatment on the weight of the animals over the course of the experiment (*F* 2,21 = 1.628, NS). A 3-way ANOVA revealed a significant main effect of session (*F* 3, 63) = 95.1, *p* < 0.0001), trial (*F* 4,84) = 32.0, *p* < 0.0001) and an interaction between session and trial (*F* 12, 252) = 14.8, *p* < 0.0001) (Fig. 1a), reflecting learning among all groups. Individual 2-way ANOVAs for each session revealed that there were no significant differences in the measured escape latencies between the different treatment groups at baseline (*F* 2,21 = 0.027, NS). However, there were significant differences in learning performance among treatment groups in the following sessions: Session 2 (*F* 2,21 = 6.101, *p* < 0.01), Session 3 (*F* 2,21 = 5.641, *p* < 0.05) and Session 4 (*F* 2,21 = 5.506, *p* < 0.05). Specially, there was a significant decrease in escape latency for the 17.4 mg flavonoid supplemented group in comparison to control for session 2, 3 and 4 (*p* < 0.05), whilst the lower dose of 8.7 mg flavonoid group was significantly faster at sessions 2 and 3 (*p* < 0.05) and marginally significant on the last session (*p* = 0.07) (Fig. 1a). On average all animals experienced a significant decrease (approximately ten-fold) in escape latency between test session 1 and test session 4 (Fig. 1a), indicating that all groups successfully acquired the spatial task reaching minimum escape latency by session 4 (*F* 3, 84 = 169.5, *p* < 0.0001) (Fig. 1a). Swimming speed was also measured in addition to latency to find the platform, revealing no significant (*p* > 0.05) differences among groups during the acquisition of the task. Importantly, this eliminates the possibility of latency results being masked by different swimming speeds among the groups. Effective consolidation of the task was assessed using a probe trial 24 h after the 4th and final testing session (Fig. 1b). A one-way ANOVA demonstrated a significant difference in time spent in the target quadrant between treatment groups (*F* 2, 23 = 3.656, *p* < 0.05). Further post-hoc tests confirmed that the 17.4 mg flavonoid group exhibited significantly enhanced recall of the platform location (*p* < 0.05) in comparison with control animals, whilst the 8.7 mg flavonoid group showed a trend for increased recall relative to control animals (*p* = 0.1) (Fig. 1b).

### 3.2 Modulation of PSA-NCAM in the dentate gyrus of the hippocampus

**Fig. 2:**
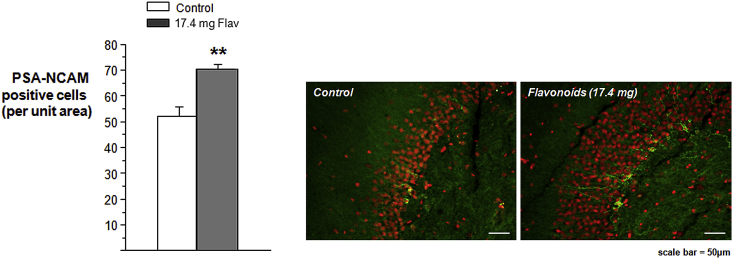
Levels of PSA-NCAM in the Dentate Gyrus of the Hippocampus. Levels of polysialylated NCAM cell frequency in the dentate gyrus of the hippocampus of adult Wistar Rats after 3 weeks of flavonoid supplementation. Animals treated with 17.4 mg of flavonoids (*n* = 5) showed a significant increase in the frequency of PSA-NCAM cells compared to animals on the control diet (*n* = 4) (**p < 0.01). Illustrative qualitative images of PSA immunoreactivity in the dentate granule cell layer/hilar border (GCL) at −5.6 mm with respect to bregma are presented (1 animal from the control group and 1 animal from the 17.4 mg flavonoid group). The green staining indicates the position of the immunostained cells at the infragranular zone and the scale bar represents 50 μm. Cell identification was aided by the use of the nuclear counter-stain propidium iodide (red). Cell counts were standardized to unit area of granule cell layer. (For interpretation of the references to color in this figure legend, the reader is referred to the web version of this article.)

PSA-NCAM expression in the adult dentate gyrus was found to be primarily associated with granule cell bodies located at the infragranular zone and their dendritic arbor that extended through the granular cell layer and into the molecular layer (Fig. 2; white arrows). Animals supplemented with 17.4 mg of flavonoids exhibited a significant increase in the number of NCAM-polysialylated cells present in the DG compared to animals on a standard diet (*t*7 = 4.49, *p* < 0.01). PSA-NCAM expression in the DG was highly correlated with both the acquisition of the memory task (Session 2: *R* = 0.87; *p* < 0.01: Session 3: *R* = 0.79, *p* < 0.01; Session 4: *R* = 0.75, *p* < 0.05) and the 24 h recall of the platform location (*R* = 0.82, *p* < 0.01).

### 3.3 Regulation of NMDA and AMPA receptors in the hippocampus

**Fig. 3:**
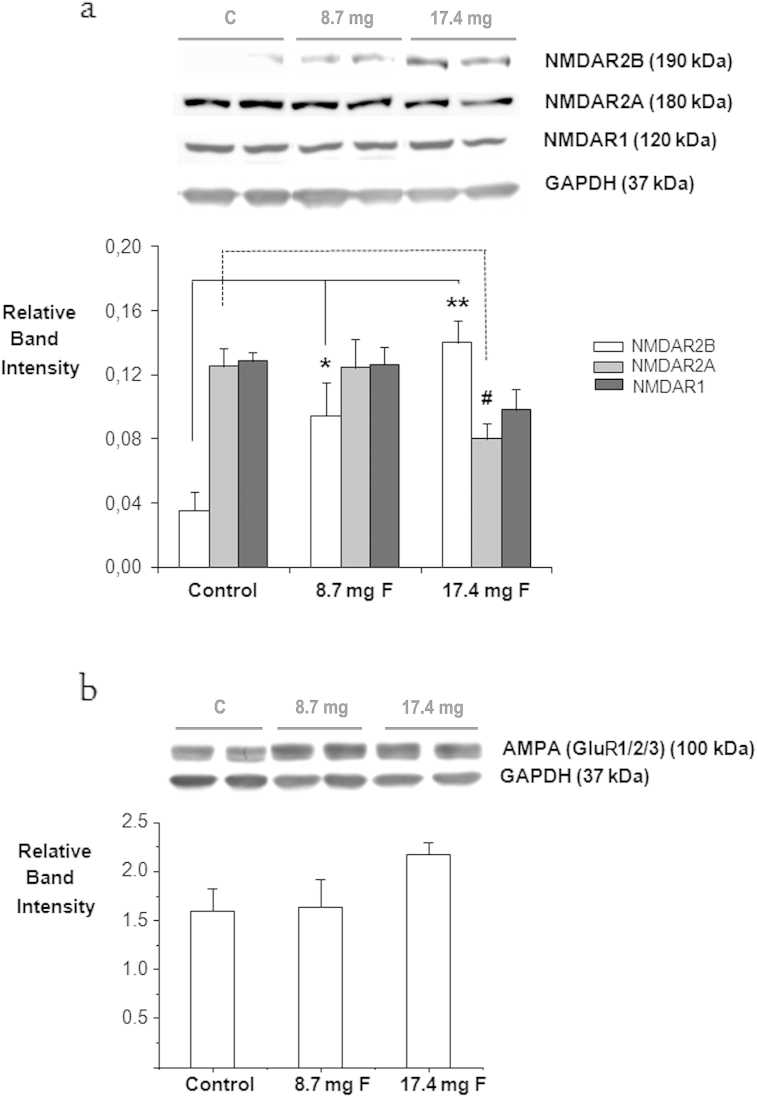
Levels of hippocampal NMDA and AMPA receptors. Hippocampal lysates were immunoblotted with antibodies to detect (a) NMDAR2B, NMDAR2A and NMDAR1; (b) AMPA (GluR 1/2/3). * Indicates a significant increase in NMDAR2B of animals supplemented with 8.7 mg of flavonoids relative to animals supplemented with control solution, *p* < 0.05; *n* = 6. *** Indicates a significant increase in NMDAR2B of animals supplemented with 17.4 mg of flavonoids relative to control animals, *p* < 0.001; *n* = 6. ^#^ Indicates a trend toward a decrease in levels of NMDAR2A of animals supplemented with 17.4 mg of flavonoids relative to control animals, *p* = 0.08, *n* = 6. GAPDH was used as loading control to normalize total levels of NMDAR2B, NMDAR2A, NMDAR1 and GluR1/2/3. Representative blots showing, left to right, protein levels in two control animals, two animals supplemented with 8.7 mg of flavonoids and two animals supplemented with 17.4 mg of flavonoids are presented.

Flavonoid intervention induced highly significant increases in the levels of the NR2B subunit (*F*2,17 = 10.843, *p* < 0.001), for both the 8.7 mg dose (*p* < 0.05) and the 17.4 mg dose (*p* < 0.001). We observe simultaneously a significant decrease in the levels of NR2A subunit after treatment (*F*2,16 = 3.804, *p* < 0.05) driven mainly by the 17.4 mg dose (*p* = 0.08). No significant changes were detected in the NR1 subunit (*F*2,17 = 2.759, NS) (Fig. 3a). Increases in NMDAR2B subunit levels were significantly correlated with the recall of the platform location in behavioral tasks (*R* = 0.48, *p* < 0.05). Furthermore, the level of NR2B subunit was highly correlated with PSA-NCAM positive cells in the dentate gyrus (*R* = 0.75, *p* < 0.05) and levels hippocampal Arc (*R* = 0.78, *p* < 0.01), outlined below. In contrast to NMDAR levels, the flavonoid intervention had no effect on the overall hippocampal levels of GluR1/2/3 receptors (*F* 2,17 = 2.003, NS) (Fig. 3b). Nonetheless, the AMPA antibody used detects simultaneously GluR1/2 and 3 which could potentially mask potential changes in individual subunits.

### 3.4 Modulation of hippocampal ERK, CREB and BDNF

**Fig. 4:**
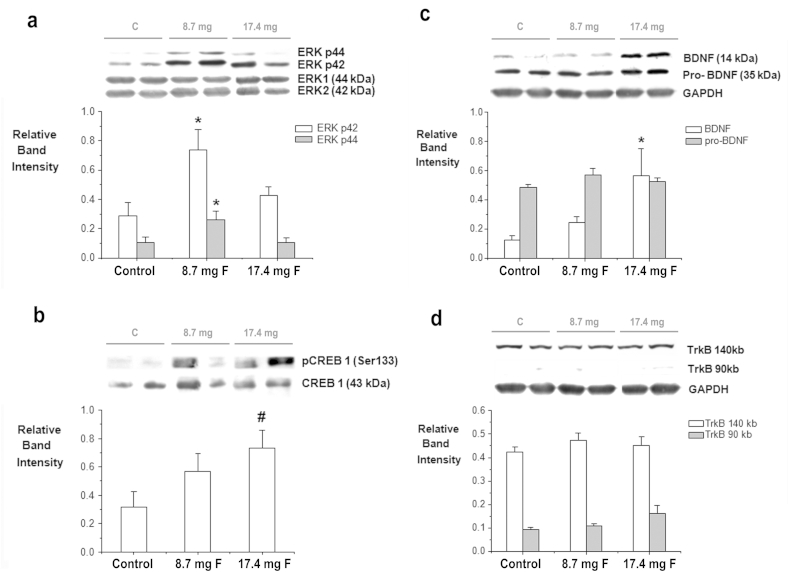
Levels of hippocampal ERK1/2 and CREB phosphorylation and total levels of hippocampal BDNF, pro-BDNF and TrkB. Hippocampal lysates were immunoblotted with antibodies to detect: (a) ERK1/2 when dually phosphorylated and total ERK. *Indicates a significant increase in phosphorylation levels of pERK1 (44 kb) and pERK2 (42 kb) of animals supplemented with 8.7 mg of flavonoids relative to animals supplemented with the control (*p* < 0.05, *n* = 6). (b) phosphorylated CREB (Ser^133^) and Total CREB. ^#^ indicates a trend for an increase of 17.4 mg flavonoid dose in relation to the control solution (*p* < 0.1, *n* = 6). CREB and ERK phosphorylation were normalized against total levels of CREB and ERK respectively. (c) Total levels of pro-BDNF (grey bars) and mature BDNF (white bars). * Indicates a significant increase in total levels of BDNF of animals supplemented with 17.4 mg of flavonoids in relation to control animals (*p* < 0.05, *n* = 6). (d) Total levels of TrkB (90 kb, 140 kb). GAPDH was used as loading control to normalize total levels BDNF, pro-BDNF and TrkB. Representative blots showing, left to right, protein levels in two control animals, two animals supplemented with 8.7 mg of flavonoids and two animals supplemented with 17.4 mg of flavonoids are presented.

The activation of the mitogen-activated protein kinase ERK1/2 was probed using a phospho-specific antibody that recognizes both phosphorylated motifs pTEpY within activated ERK1/2. Hippocampal levels of phospho-ERK1 and phospho-ERK2 were significantly regulated by the flavonoid intervention (*F*2,17 = 4.435, *p* < 0.05 and *F*2,17 = 4.849, *p* < 0.05, respectively). Subsequent post-hoc tests, revealed a significant increase in pERK1/2 induced by the 8.7 mg dose (*p* < 0.05) but not the 17.4 mg dose (NS) (Fig. 4a). As ERK1/2 are known to mediate the activation of CREB1 at Ser^133^, we also probed changes in CREB activation using a phospho-specific antibody that recognizes CREB when phosphorylated at the Ser^133^ residue. There was a trend towards an increase in the levels of activated CREB for flavonoid animals in comparison with control (*F*2,17 = 2.965, *p* = 0.09), with increases in pCREB1 being dose-dependent in nature: 17.4 mg dose (*p* = 0.07), 8.7 mg dose (NS) (Fig. 4b). A significant increase in the levels of mature BDNF was observed in response to flavonoid supplementation (*F*2,17 = 4.217, *p* < 0.05), which again was greater with the 17.4 mg dose (*p* < 0.05) (Fig. 4c). No changes were found between diet groups for pro-BDNF (*F*2,17 = 1.645, NS) (Fig. 4c) or for total levels of the TrkB receptor (*F*2,17 = 2.885, NS; *F*2,17 = 0.639, NS) (Fig. 4d).

### 3.5 Regulation of Akt, mTOR and Arc/Arg3.1 in the hippocampus

**Fig. 5:**
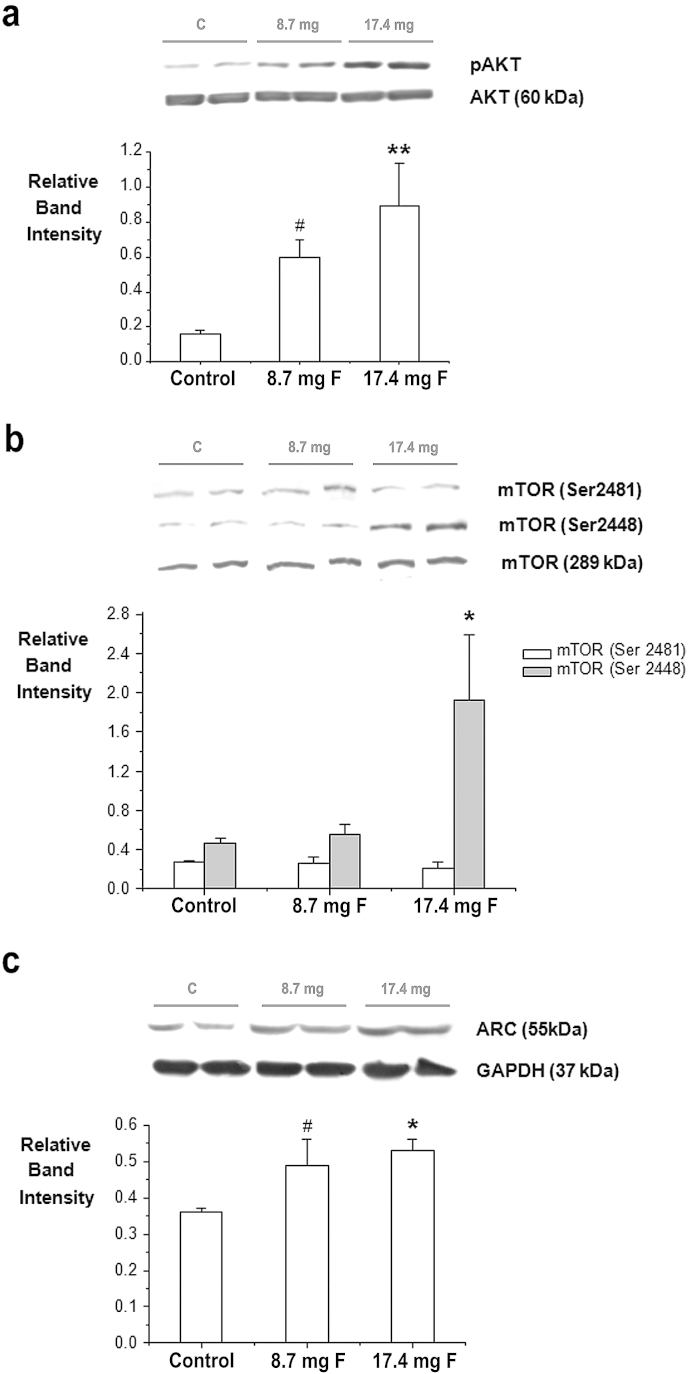
Levels of hippocampal AKT and mTOR phosphorylation and Arc/Arg3.1. Hippocampal lysates were immunoblotted with antibodies to detect: (a) Akt when phosphorylated at Ser^473^ and total levels of Akt. ** Indicates a significant increase in phosphorylation levels of Akt in 17.4 mg supplemented animals relative to animals supplemented with a control solution (*p* < 0.01, *n* = 6). # Indicates a trend toward increase for animals supplemented with 8.7 mg of flavonoids (*p* ≤ 0.1, *n* = 6); (b) mTOR when phosphorylated at Ser ^2448^ (grey bars) and at Ser ^2481^ (white bars). * Indicates a significant increase in phosphorylation levels of mTOR at Ser^2448^ in 17.4 mg supplemented animals relative to animals supplemented with control solution (*p* < 0.05, *n* = 6); Akt and mTOR phosphorylation were normalized against total levels of Akt and mTOR respectively. (c) Total levels of Arc/Arg3.1. *Indicates a significant increase in total levels of Arc/Arg3.1 in 17.4 mg flavonoid supplemented animals relative to animals supplemented with control solution (*p* < 0.05, *n* = 6); # Indicates a trend towards an increase for animals supplemented with 8.7 mg of flavonoids (*p* ≤ 0.1, *n* = 6). GAPDH was used as loading control to normalize total levels of Arc/Arg3.1. Representative blots showing, left to right, protein levels in two control animals, two animals supplemented with 8.7 mg of flavonoids and two animals supplemented with 17.4 mg of flavonoids are presented.

Flavonoid intervention had a significant impact on the activation of Akt (*F* 2,17 = 5.573, *p* < 0.05), primarily at the 17.4 mg dose (*p* < 0.01), whereas the 8.7 mg dose showed a trend to increase (*p* < 0.1) (Fig. 5a). Furthermore, flavonoid intervention was observed to significantly induce mTOR phosphorylation at the Ser^2448^ residue (*F* 2,17 = 4.459, *p* < 0.05) but not the Ser^2481^ residue (*F*2,17 = 0.291, NS), although these changes only manifested at the 17.4 mg intervention dose (*p* < 0.05) (Fig. 5b). The activity-regulated cytoskeleton-associated protein Arc/Arg3.1 was also significantly increased by flavonoid intervention in comparison to the control group (*F* 2,17 = 3.788, *p* < 0.05) (Fig. 5c). The pattern of Arc activation was similar to that observed for other molecular parameters, with the 17.4 mg dose inducing a significant increase in Arc/Arg3.1 (*p* < 0.05) and the 8.7 mg dose showing a trend for an increase in Arc (*p* = 0.1) (Fig. 5c). The increases in hippocampal Arc following flavonoid administration were found to be highly correlated with the levels of hippocampal NR2B subunit receptor (*R* = 0.78; *p* < 0.01).

## 4 Discussion

Research into the impact of flavonoid-rich foods on memory, learning and cognitive performance has primarily focused on their potential to reverse cognitive deficits in aged animals (Casadesus et al., 2004; Li et al., 2009a,b) or transgenic mouse models of neurodegenerative disease, such as Alzheimer Disease (Joseph et al., 2003). In the present study, we show that a 3-week supplementation with 8.7 mg or 17.4 mg of flavonoids per day (containing both anthocyanins and flavanols) (Table 1) is also effective in improving spatial learning and memory in healthy, young animals. Both doses (8.7 mg and 17.4 mg), which broadly reflect a dietary level of intervention, were equally efficacious in enhancing memory acquisition, with the 17.4 mg dose being more effective toward memory recall, 24 post testing, which is typically more demanding. The observed flavonoid-induced improvements in behavior were associated with specific changes in protein expression in the hippocampus, in particular 24 h recall was found to be highly correlated with hippocampal levels of the NR2B subunit of the NMDA receptor and with the levels of the adhesion molecule PSA-NCAM in the DG of the hippocampus, both proteins linked to efficient and persistent LTP and spatial learning (Gascon et al., 2007b; Li et al., 2007; Murphy et al., 2006; Sandi, 2004).

Regarding the establishment of a causal link between flavonoid intake and neuronal function, we have shown previously that both anthocyanins and flavanols are quantifiable in the hippocampus after ingestion, establishing the presence of the potential active compounds at the site of action in the time frame of the behavioral effect (Williams et al., 2008). We have further shown that pure flavanols and anthocyanins when administered separately result equally in improvements in learning and memory as well as in modulation of BDNF levels in the hippocampus, strongly suggesting that flavonoids are the active components driving the beneficial effects of flavonoid-rich foods in brain function (Rendeiro et al., 2013). The present data supports our recent study in young animals showing that a 7-week intervention with blueberry resulted in significant improvements in spatial memory, along with BDNF regulation in the hippocampus (Rendeiro et al., 2012). In the present study we show an effect on spatial memory after only a 3 week intervention on an MWM learning paradigm, which allowed us to distinguish the effect of flavonoids on both acquisition and consolidation aspects of learning. Notably, the data emanating from this study suggests a novel mechanism by which flavonoids may act in the brain and it shows a dose response at both behavioral and molecular levels after a 3-week administration of dietary amounts of flavonoids. This further adds to the causality criteria for the assessment of flavonoids as potential mediators of brain function.

PSA-NCAM plays an important role during brain development, although its expression persists during adulthood in brain structures such as the hippocampus that display a high degree of plasticity (Gascon et al., 2007b). The negatively charged PSA chain of NCAM has been suggested to act as a spacer, decreasing NCAM–NCAM mediated cell adhesion between neurons, therefore facilitating structural remodeling and consequently promoting activity-induced plasticity (Kochlamazashvili et al., 2010; Rutishauser, 2008; Rutishauser and Landmesser, 1996). Furthermore, the polysialylation of NCAM in the DG of the hippocampus is known to support the development of basal synaptic transmission in this region (Stoenica et al., 2006) and has been reported to be learning-specific, in particular during spatial learning tasks (Murphy et al., 1996; Venero et al., 2006). In addition to the regulation of adhesion strength, there is evidence that the polysialylation of NCAM may also allow it to regulate the activation of signaling pathways linked to the control of synaptic plasticity (Dityatev et al., 2004; Kiss et al., 2001; Muller et al., 2000). Although, the exact mechanisms are unclear, there is evidence to suggest that regulation of NCAM polysialylation is linked to BDNF signaling, since defective LTP observed in PSA-NCAM-deficient hippocampus can be selectively rescued by BDNF (as well as exogenous application of PSA residues or recombinant PSA-NCAM) and is associated with a reduced activation of BDNF signaling (Muller et al., 2000). In agreement with this we observe significant increases in BDNF levels in the hippocampus of flavonoid fed rats, suggesting a potential mechanistic link between PSA-NCAM and BDNF-associated signaling in flavonoid-induced memory improvements.

The observed increases in BDNF levels in flavonoid supplemented animals appear to be linked to the activation of ERK/CREB signaling in our animals, since BDNF is an important CREB target involved in memory and learning events (Ying et al., 2002). Indeed, there is evidence that NCAM intrinsic signaling results in MAPK activation (via Fyn-FAK-Ras) (Kolkova et al., 2000). Furthermore, previous studies have demonstrated that activation of NCAM by PSA may induce the activation of CREB (Aonurm-Helm et al., 2008). On the other hand, BDNF, once released into the synapse, triggers the activation of the PI3 kinase/Akt signaling pathway and further activation of mTOR pathway through its binding to TrkB receptors (Kumar et al., 2005; Takei et al., 2004). Although we observed no increase in the total levels of TrkB, we observed activation of Akt and selective phosphorylation of mTOR at Ser ^2448^ in flavonoid fed animals, suggesting increased BDNF binding. Such events are specifically involved in the regulation of protein translation (Bekinschtein et al., 2007) and there is strong evidence suggesting that the Akt/mTOR and the MAPK (ERK1/2) pathways act in parallel/coordination to regulate morphological changes in neuronal dendrites (Dijkhuizen and Ghosh, 2005; Kumar et al., 2005; Rodgers and Theibert, 2002). Moreover, Arc, an important Akt/mTOR target, is involved in the regulation of cytoskeletal actin and impact on dendritic morphogenesis and spine formation, events regarded as being pivotal in synaptic plasticity associated with learning (Messaoudi et al., 2007). These data agree with our previous observations that blueberry induced behavioral changes in old animals are underpinned by increases in hippocampal BDNF and parallel activation of ERK/CREB and Akt/mTOR/Arc pathways (Williams et al., 2008).

Alterations in NMDAR activation have also been postulated to be regulated via BDNF, since manipulations that reduce BDNF expression in the hippocampus also reduce NMDA receptor subunit expression (Roceri et al., 2002). In particular, high levels of NR2B-containing NMDA receptors confer distinct gating and pharmacological properties to the receptor channel that impact on the kinetics of receptor activation facilitating glutamate signaling (Cull-Candy et al., 2001). This can dramatically alter an animal's capacity to exhibit LTP contributing to enhanced synaptic plasticity that results in memory and learning improvements (Farmer et al., 2004; Tang et al., 1999; Vasuta et al., 2007). Our observations of an increase in NR2B-NMDA receptors in the hippocampus of flavonoid supplemented rats suggest that flavonoids may alter glutamatergic signaling and consequently affect memory. In support of this, our simultaneous observation of a trend towards a decrease on the levels of the subunit NR2A, suggests a replacement of NR2A by NR2B subunits in the NMDA heteromeric receptor assemblies, which is known to favor LTP (Foster et al., 2010). Indeed changes in spatial memory performance induced by flavonoid intervention significantly correlated with hippocampal levels of the NR2B glutamate receptor subunit. It has been reported previously that animals fed with blueberry for 6–8 weeks ameliorate age-related declines in NMDA receptor-dependent LTP in the CA1 region of the hippocampus, suggesting that intervention with blueberry flavonoids increases NMDAR function and enhances glutamatergic signaling (Coultrap et al., 2008). However, since this was conducted in a resting state (not after a learning paradigm), there is no direct evidence that the changes in LTP observed translate into memory improvements after flavonoid consumption.

Finally, the regulation of PSA-NCAM expression by NMDAR activation has been described in several systems, suggesting a functional link between these two proteins (Bouzioukh et al., 2001; Butler et al., 1999; Dityatev et al., 2004). Although we observed a good correlation between the increases in PSA-NCAM and NR2B hippocampal levels following supplementation with flavonoids, we cannot at this stage conclude to what extent these two events are linked and their relative contribution to the observed behavioral outcomes. Interestingly, increased levels of PSA-NCAM are a typical feature of newly generated neurons in the dentate gyrus of the hippocampus (Garcia-Verdugo et al., 1998; Rousselot et al., 1995) and this has been suggested to play an important role in the survival rate of such neurons by facilitating their integration into functional circuits (Aimone et al., 2006; Gascon et al., 2007a). Indeed, there is strong evidence suggesting that processes that enhance synaptic plasticity, such as spatial learning, increase the survival of newborn neurons and the efficiency of integration of these in hippocampal circuitry (Drapeau et al., 2007; Dupret et al., 2007). Although, we are presently unable to conclude the extent to which a flavonoid-rich diet may affect specifically the plasticity of newborn neurons, future research should further explore this by looking at survival and integration rates of immature neurons following flavonoid supplementation (Seki, 2002; von Bohlen Und Halbach, 2007).

In summary, the improvements in memory and learning observed in flavonoid fed young animals are likely to be associated with flavonoid-induced polysialylation of NCAM. In addition to this, a parallel elevation of NR2B-containing NMDA receptor at synaptic sites, suggests an enhancement of glutamate signaling, potentially prolonged NMDAR currents and more stable LTP. Our data further suggest that such events might be linked by sustained activation of the signaling ERK/CREB/BDNF and Akt/mTOR/Arc pathways. These flavonoid-induced changes at the neuronal level are likely to account, at least partially, for the observed improvements in spatial memory in young animals following flavonoid supplementation. Nonetheless, in order to fully establish a causal relationship between flavonoid intake and brain function, future work will focus on showing that the withholding of flavonoid consumption results in a reversal or attenuation of the behavioral effects. Furthermore the establishment of the mechanism of action will require the inhibition of the relevant mediator pathways/receptors, (e.g. NMDA receptor and NCAM polysialylation) resulting in a loss or attenuation of learning following flavonoid intake. Overall, we should bear in mind that we cannot at this stage draw conclusions regarding whether flavonoids are triggering such effects by acting centrally or whether these effects are mediated by peripheral actions (e.g vascular related-effects).

## Author disclosure statement

The authors declare no competing financial interests.

## Figures and Tables

**Table 1:** Characterization of the flavonoid profile administered to young rats for a period of 3 weeks. The quantification of levels of both anthocyanins and flavanols oligomers showed that *Dose I* delivered a total of 8.71 mg of flavonoids per animal per day, consisting of 5.37 mg of anthocyanins and 3.34 mg of flavanols; whilst *Dose II* delivered a total 17.42 mg of flavonoids per animal per day, consisting of 10.75 mg of anthocyanins and 6.67 mg of flavanols.

Amounts per day per animal	Dose I	Dose II
*Anthocyanins (mg)*		
Delphinidin	2.01	4.02
Cyanidin	0.37	0.73
Petunidin	1.04	2.08
Peonidin	0.08	0.16
Malvidin	1.89	3.76

*Total Anthocyanins*	*5.37*	*10.75*

*Flavanols (mg)*		
Monomers	0.29	0.58
Dimers	0.96	1.93
Oligomers (3–10)	2.08	4.16

*Total Flavanols*	*3.34*	*6.67*

Total Flavonoids	8.71	17.42

## List of abbreviations

PSA: polysialylated acid
NCAM: neural cell adhesion molecule
NMDA-R: *N*-methyl-d-aspartate receptor
AMPA: α-amino-3-hydroxy-5-methyl-4-isoxazolepropionic acid
MAPK: mitogen-activated protein kinase
ERK: extracellular signal-regulated protein kinase
PI3K: phosphatidylinositol-3 kinase
CREB: cAMP-response element binding protein
mTOR: mammalian target of rapamycin
BDNF: brain-derived neurotrophic factor
Arc/Arg3.1: activity-regulated cytoskeleton-associated protein
GAPDH: glyceraldehyde-3-phosphate dehydrogenase
DG: Dentate Gyrus
SPSS: Statistical Product and Service Solutions.

## References

[bib1] Aimone J.B., Wiles J., Gage F.H. (2006). Potential role for adult neurogenesis in the encoding of time in new memories. Nat. Neurosci..

[bib2] Aonurm-Helm A., Zharkovsky T., Jurgenson M., Kalda A., Zharkovsky A. (2008). Dysregulated CREB signaling pathway in the brain of neural cell adhesion molecule (NCAM)-deficient mice. Brain Res..

[bib3] Becker C.G., Artola A., Gerardy-Schahn R., Becker T., Welzl H., Schachner M. (1996). The polysialic acid modification of the neural cell adhesion molecule is involved in spatial learning and hippocampal long-term potentiation. J. Neurosci. Res..

[bib4] Bekinschtein P., Katche C., Slipczuk L.N., Igaz L.M., Cammarota M., Izquierdo I. (2007). mTOR signaling in the hippocampus is necessary for memory formation. Neurobiol. Learn. Memory.

[bib5] Benson D.L., Schnapp L.M., Shapiro L., Huntley G.W. (2000). Making memories stick: cell-adhesion molecules in synaptic plasticity. Trends Cell Biol..

[bib6] Bouzioukh F., Tell F., Jean A., Rougon G. (2001). NMDA receptor and nitric oxide synthase activation regulate polysialylated neural cell adhesion molecule expression in adult brainstem synapses. J. Neurosci..

[bib7] Butler A.K., Uryu K., Rougon G., Chesselet M.F. (1999). *N*-methyl-d-aspartate receptor blockade affects polysialylated neural cell adhesion molecule expression and synaptic density during striatal development. Neuroscience.

[bib8] Casadesus G., Shukitt-Hale B., Stellwagen H.M., Zhu X., Lee H.G., Smith M.A. (2004). Modulation of hippocampal plasticity and cognitive behavior by short-term blueberry supplementation in aged rats. Nutr. Neurosci..

[bib9] Coultrap S.J., Bickford P.C., Browning M.D. (2008). Blueberry-enriched diet ameliorates age-related declines in NMDA receptor-dependent LTP. Age (Dordr).

[bib10] Cull-Candy S., Brickley S., Farrant M. (2001). NMDA receptor subunits: diversity, development and disease. Curr. Opin. Neurobiol..

[bib11] Cull-Candy S.G., Leszkiewicz D.N. (2004). Role of distinct NMDA receptor subtypes at central synapses. Sci. STKE.

[bib12] Dijkhuizen P.A., Ghosh A. (2005). BDNF regulates primary dendrite formation in cortical neurons via the PI3-kinase and MAP kinase signaling pathways. J. Neurobiol..

[bib13] Dinges D.F. (2006). Cocoa flavanols, cerebral blood flow, cognition, and health: going forward. J. Cardiovasc. Pharmacol..

[bib14] Dityatev A., Dityateva G., Sytnyk V., Delling M., Toni N., Nikonenko I. (2004). Polysialylated neural cell adhesion molecule promotes remodeling and formation of hippocampal synapses. J. Neurosci..

[bib15] Doyle E., Nolan P.M., Bell R., Regan C.M. (1992). Intraventricular infusions of anti-neural cell adhesion molecules in a discrete posttraining period impair consolidation of a passive avoidance response in the rat. J. Neurochem..

[bib16] Drapeau E., Montaron M.F., Aguerre S., Abrous D.N. (2007). Learning-induced survival of new neurons depends on the cognitive status of aged rats. J. Neurosci..

[bib17] Dupret D., Fabre A., Dobrossy M.D., Panatier A., Rodriguez J.J., Lamarque S. (2007). Spatial learning depends on both the addition and removal of new hippocampal neurons. PLoS Biol..

[bib18] Farmer J., Zhao X., van Praag H., Wodtke K., Gage F.H., Christie B.R. (2004). Effects of voluntary exercise on synaptic plasticity and gene expression in the dentate gyrus of adult male Sprague-Dawley rats in vivo. Neuroscience.

[bib19] Foster K.A., McLaughlin N., Edbauer D., Phillips M., Bolton A., Constantine-Paton M. (2010). Distinct roles of NR2A and NR2B cytoplasmic tails in long-term potentiation. J. Neurosci..

[bib20] Fox G.B., Kennedy N., Regan C.M. (1995). Polysialylated neural cell adhesion molecule expression by neurons and astroglial processes in the rat dentate gyrus declines dramatically with increasing age. Int. J. Dev. Neurosci..

[bib21] Garcia-Verdugo J.M., Doetsch F., Wichterle H., Lim D.A., Alvarez-Buylla A. (1998). Architecture and cell types of the adult subventricular zone: in search of the stem cells. J. Neurobiol..

[bib22] Gascon E., Vutskits L., Jenny B., Durbec P., Kiss J.Z. (2007). PSA-NCAM in postnatally generated immature neurons of the olfactory bulb: a crucial role in regulating p75 expression and cell survival. Development.

[bib23] Gascon E., Vutskits L., Kiss J.Z. (2007). Polysialic acid-neural cell adhesion molecule in brain plasticity: from synapses to integration of new neurons. Brain Res. Rev..

[bib24] Hammond M.S., Sims C., Parameshwaran K., Suppiramaniam V., Schachner M., Dityatev A. (2006). Neural cell adhesion molecule-associated polysialic acid inhibits NR2B-containing N-methyl-d-aspartate receptors and prevents glutamate-induced cell death. J. Biol. Chem..

[bib25] Haque A.M., Hashimoto M., Katakura M., Tanabe Y., Hara Y., Shido O. (2006). Long-term administration of green tea catechins improves spatial cognition learning ability in rats. J. Nutr..

[bib26] Joseph J.A., Denisova N.A., Arendash G., Gordon M., Diamond D., Shukitt-Hale B. (2003). Blueberry supplementation enhances signaling and prevents behavioral deficits in an Alzheimer disease model. Nutr. Neurosci..

[bib27] Joseph J.A., Shukitt-Hale B., Denisova N.A., Bielinski D., Martin A., McEwen J.J. (1999). Reversals of age-related declines in neuronal signal transduction, cognitive, and motor behavioral deficits with blueberry, spinach, or strawberry dietary supplementation. J. Neurosci..

[bib28] Kaur T., Pathak C.M., Pandhi P., Khanduja K.L. (2008). Effects of green tea extract on learning, memory, behavior and acetylcholinesterase activity in young and old male rats. Brain Cogn..

[bib29] Kim M.J., Dunah A.W., Wang Y.T., Sheng M. (2005). Differential roles of NR2A- and NR2B-containing NMDA receptors in Ras-ERK signaling and AMPA receptor trafficking. Neuron.

[bib30] Kiss J.Z., Troncoso E., Djebbara Z., Vutskits L., Muller D. (2001). The role of neural cell adhesion molecules in plasticity and repair. Brain Res. Brain Res. Rev..

[bib31] Kochlamazashvili G., Bukalo O., Senkov O., Salmen B., Gerardy-Schahn R., Engel A.K. (2012). Restoration of synaptic plasticity and learning in young and aged NCAM-deficient mice by enhancing neurotransmission mediated by GluN2A-containing NMDA receptors. J. Neurosci..

[bib32] Kochlamazashvili G., Senkov O., Grebenyuk S., Robinson C., Xiao M.F., Stummeyer K. (2010). Neural cell adhesion molecule-associated polysialic acid regulates synaptic plasticity and learning by restraining the signaling through GluN2B-containing NMDA receptors. J. Neurosci..

[bib33] Kolkova K., Novitskaya V., Pedersen N., Berezin V., Bock E. (2000). Neural cell adhesion molecule-stimulated neurite outgrowth depends on activation of protein kinase C and the Ras-mitogen-activated protein kinase pathway. J. Neurosci..

[bib34] Krikorian R., Shidler M.D., Nash T.A., Kalt W., Vinqvist-Tymchuk M.R., Shukitt-Hale B. (2010). Blueberry supplementation improves memory in older adults. J. Agric. Food Chem..

[bib35] Kromhout D., Menotti A., Kesteloot H., Sans S. (2002). Prevention of coronary heart disease by diet and lifestyle: evidence from prospective cross-cultural, cohort, and intervention studies. Circulation.

[bib36] Kumar V., Zhang M.X., Swank M.W., Kunz J., Wu G.Y. (2005). Regulation of dendritic morphogenesis by Ras-PI3K-Akt-mTOR and Ras-MAPK signaling pathways. J. Neurosci..

[bib37] Li Q., Zhao H.F., Zhang Z.F., Liu Z.G., Pei X.R., Wang J.B. (2009). Long-term administration of green tea catechins prevents age-related spatial learning and memory decline in C57BL/6 J mice by regulating hippocampal cyclic amp-response element binding protein signaling cascade. Neuroscience.

[bib38] Li Q., Zhao H.F., Zhang Z.F., Liu Z.G., Pei X.R., Wang J.B. (2009). Long-term green tea catechin administration prevents spatial learning and memory impairment in senescence-accelerated mouse prone-8 mice by decreasing Abeta1-42 oligomers and upregulating synaptic plasticity-related proteins in the hippocampus. Neuroscience.

[bib39] Li R., Huang F.S., Abbas A.K., Wigstrom H. (2007). Role of NMDA receptor subtypes in different forms of NMDA-dependent synaptic plasticity. BMC Neurosci..

[bib40] Li W., Keifer J. (2009). BDNF-induced synaptic delivery of AMPAR subunits is differentially dependent on NMDA receptors and requires ERK. Neurobiol. Learn. Memory.

[bib41] Lopez-Fernandez M.A., Montaron M.F., Varea E., Rougon G., Venero C., Abrous D.N. (2007). Upregulation of polysialylated neural cell adhesion molecule in the dorsal hippocampus after contextual fear conditioning is involved in long-term memory formation. J. Neurosci..

[bib42] MacDonald J.F., Jackson M.F., Beazely M.A. (2006). Hippocampal long-term synaptic plasticity and signal amplification of NMDA receptors. Crit. Rev. Neurobiol..

[bib43] Malinow R., Malenka R.C. (2002). AMPA receptor trafficking and synaptic plasticity. Annu. Rev. Neurosci..

[bib44] Messaoudi E., Kanhema T., Soule J., Tiron A., Dagyte G., da Silva B. (2007). Sustained Arc/Arg3.1 synthesis controls long-term potentiation consolidation through regulation of local actin polymerization in the dentate gyrus in vivo. J. Neurosci..

[bib45] Muller D., Djebbara-Hannas Z., Jourdain P., Vutskits L., Durbec P., Rougon G. (2000). Brain-derived neurotrophic factor restores long-term potentiation in polysialic acid-neural cell adhesion molecule-deficient hippocampus. Proc. Natl. Acad. Sci. U.S.A..

[bib46] Muller D., Wang C., Skibo G., Toni N., Cremer H., Calaora V. (1996). PSA-NCAM is required for activity-induced synaptic plasticity. Neuron.

[bib47] Murphy K.J., Foley A.G., O'Connell A.W., Regan C.M. (2006). Chronic exposure of rats to cognition enhancing drugs produces a neuroplastic response identical to that obtained by complex environment rearing. Neuropsychopharmacology.

[bib48] Murphy K.J., O'Connell A.W., Regan C.M. (1996). Repetitive and transient increases in hippocampal neural cell adhesion molecule polysialylation state following multitrial spatial training. J. Neurochem..

[bib49] Parrott M.D., Greenwood C.E. (2007). Dietary influences on cognitive function with aging: from high-fat diets to healthful eating. Ann. N.Y. Acad. Sci..

[bib77] Paxinos G., Watson C. (1986). The Rat Brain in Stereotaxic Coordinates.

[bib50] Perez-Otano I., Ehlers M.D. (2004). Learning from NMDA receptor trafficking: clues to the development and maturation of glutamatergic synapses. Neuro-Signals.

[bib51] Rendeiro C., Vauzour D., Kean R.J., Butler L.T., Rattray M., Spencer J.P. (2012). Blueberry supplementation induces spatial memory improvements and region-specific regulation of hippocampal BDNF mRNA expression in young rats. Psychopharmacology (Berl.).

[bib52] Rendeiro C., Vauzour D., Rattray M., Waffo-Téguo P., Mérillon J.M., Butler L.T. (2013). Dietary levels of pure flavonoids improve spatial memory performance and increase hippocampal brain-derived neurotrophic factor. PLoS One.

[bib53] Roceri M., Hendriks W., Racagni G., Ellenbroek B.A., Riva M.A. (2002). Early maternal deprivation reduces the expression of BDNF and NMDA receptor subunits in rat hippocampus. Mol. Psychiatry.

[bib54] Rodgers E.E., Theibert A.B. (2002). Functions of PI 3-kinase in development of the nervous system. Int. J. Dev. Neurosci..

[bib55] Rodriguez-Mateos A., Cifuentes-Gomez T., Tabatabaee S., Lecras C., Spencer J.P. (2012). Procyanidin, anthocyanin, and chlorogenic acid contents of highbush and lowbush blueberries. J. Agric. Food Chem..

[bib56] Ronn L.C., Berezin V., Bock E. (2000). The neural cell adhesion molecule in synaptic plasticity and ageing. Int. J. Dev. Neurosci..

[bib57] Ronn L.C., Hartz B.P., Bock E. (1998). The neural cell adhesion molecule (NCAM) in development and plasticity of the nervous system. Exp. Gerontol..

[bib58] Rousselot P., Lois C., Alvarez-Buylla A. (1995). Embryonic (PSA) N-CAM reveals chains of migrating neuroblasts between the lateral ventricle and the olfactory bulb of adult mice. J. Comp. Neurol..

[bib59] Rumpel S., LeDoux J., Zador A., Malinow R. (2005). Postsynaptic receptor trafficking underlying a form of associative learning. Science (New York, NY.

[bib60] Rutishauser U. (2008). Polysialic acid in the plasticity of the developing and adult vertebrate nervous system. Nat. Rev. Neurosci..

[bib61] Rutishauser U., Landmesser L. (1996). Polysialic acid in the vertebrate nervous system: a promoter of plasticity in cell–cell interactions. Trends Neurosci..

[bib62] Sandi C. (2004). Stress, cognitive impairment and cell adhesion molecules. Nat. Rev. Neurosci..

[bib63] Schroeter H., Bahia P., Spencer J.P., Sheppard O., Rattray M., Cadenas E. (2007). (-)Epicatechin stimulates ERK-dependent cyclic AMP response element activity and up-regulates GluR2 in cortical neurons. J. Neurochem..

[bib64] Seki T. (2002). Expression patterns of immature neuronal markers PSA-NCAM, CRMP-4 and NeuroD in the hippocampus of young adult and aged rodents. J. Neurosci. Res..

[bib65] Seymour C.M., Foley A.G., Murphy K.J., Regan C.M. (2008). Intraventricular infusions of anti-NCAM PSA impair the process of consolidation of both avoidance conditioning and spatial learning paradigms in Wistar rats. Neuroscience.

[bib66] Sheng M., Kim M.J. (2002). Postsynaptic signaling and plasticity mechanisms. Science (New York, NY).

[bib67] Spencer J.P. (2008). Flavonoids: modulators of brain function?. Br. J. Nutr..

[bib68] Stoenica L., Senkov O., Gerardy-Schahn R., Weinhold B., Schachner M., Dityatev A. (2006). In vivo synaptic plasticity in the dentate gyrus of mice deficient in the neural cell adhesion molecule NCAM or its polysialic acid. Eur. J. Neurosci..

[bib69] Takei N., Inamura N., Kawamura M., Namba H., Hara K., Yonezawa K. (2004). Brain-derived neurotrophic factor induces mammalian target of rapamycin-dependent local activation of translation machinery and protein synthesis in neuronal dendrites. J. Neurosci..

[bib70] Tang Y.P., Shimizu E., Dube G.R., Rampon C., Kerchner G.A., Zhuo M. (1999). Genetic enhancement of learning and memory in mice. Nature.

[bib71] Vasuta C., Caunt C., James R., Samadi S., Schibuk E., Kannangara T. (2007). Effects of exercise on NMDA receptor subunit contributions to bidirectional synaptic plasticity in the mouse dentate gyrus. Hippocampus.

[bib72] Vauzour D., Vafeiadou K., Rice-Evans C., Williams R.J., Spencer J.P.E. (2007). Activation of pro-survival Akt and ERK1/2 signalling pathways underlie the anti-apoptotic effects of flavanones in cortical neurons. J. Neurochem..

[bib73] Venero C., Herrero A.I., Touyarot K., Cambon K., Lopez-Fernandez M.A., Berezin V. (2006). Hippocampal up-regulation of NCAM expression and polysialylation plays a key role on spatial memory. Eur. J. Neurosci..

[bib74] von Bohlen Und Halbach O. (2007). Immunohistological markers for staging neurogenesis in adult hippocampus. Cell Tissue Res..

[bib75] Williams C.M., El Mohsen M.A., Vauzour D., Rendeiro C., Butler L.T., Ellis J.A. (2008). Blueberry-induced changes in spatial working memory correlate with changes in hippocampal CREB phosphorylation and brain-derived neurotrophic factor (BDNF) levels. Free Radic. Biol. Med..

[bib76] Ying S.W., Futter M., Rosenblum K., Webber M.J., Hunt S.P., Bliss T.V. (2002). Brain-derived neurotrophic factor induces long-term potentiation in intact adult hippocampus: requirement for ERK activation coupled to CREB and upregulation of Arc synthesis. J. Neurosci..

